# The combination of rolipram and cilostamide improved the developmental competence of cloned porcine embryos

**DOI:** 10.1038/s41598-023-32677-3

**Published:** 2023-04-07

**Authors:** Bereket Molla Tanga, Xun Fang, Seonggyu Bang, Chaerim Seo, Heejae Kang, Dabin Cha, Ahmad Yar Qamar, Joohyun Shim, Kimyung Choi, Islam M. Saadeldin, Sanghoon Lee, Jongki Cho

**Affiliations:** 1 Lab of Theriogenology, College of Veterinary Medicine, Chungnam National University, 99, Daehak-ro, Daejeon, 34134 Republic of Korea; 2 School of Veterinary Medicine, Hawassa University, Hawassa, Ethiopia; 3 College of Veterinary & Animal Science, University of Veterinary & Animal Sciences, Lahore, Pakistan; 4 Department of Transgenic Animal Research, Optipharm, Inc., Chungcheongbuk-do, Cheongju-si, Republic of Korea; 5 Research Institute of Veterinary Medicine, Chungnam National University, Daejeon, Republic of Korea

**Keywords:** Biotechnology, Developmental biology

## Abstract

In vitro maturation of porcine oocytes is characterized by asynchronous cytoplasmic and nuclear maturation, leading to less competent oocytes supporting embryo development. The purpose of this study was to evaluate the combined effect of rolipram and cilostamide as cyclic Adenine monophosphate (cAMP) modulators to find the maximum cAMP levels that temporarily arrest meiosis. We determined the optimal time to maintain functional gap junction communication during pre-in vitro maturation to be four hours. Oocyte competence was evaluated by the level of glutathione, reactive oxygen species, meiotic progression, and gene expression. We evaluated embryonic developmental competence after parthenogenetic activation and somatic cell nuclear transfer. The combined treatment group showed significantly higher glutathione and lower reactive oxygen species levels and a higher maturation rate than the control and single treatment groups. Cleavage and blastocyst formation rates in parthenogenetic activation and somatic cell nuclear transfer embryos were higher in two-phase in vitro maturation than in the other groups. The relative levels of *BMP15*and *GDF9* expression were increased in two-phase in vitro maturation. Somatic cell nuclear transfer blastocysts from two-phase in vitro maturation oocytes showed a lower level of expression of apoptotic genes than the control, indicating better pre-implantation developmental competence. The combination of rolipram and cilostamide resulted in optimal synchrony of cytoplasmic and nuclear maturation in porcine in vitro matured oocytes and there by enhanced the developmental competence of pre-implantation embryos.

## Introduction

Porcine in vitro production (IVP) is a tool used to create suitable animal models to study human diseases or to develop bio-organs as donors for xenotransplantation due to the relatively high physiological similarity between humans and porcine^1^. The IVP of porcine embryos is usually performed by somatic cell nuclear transfer (SCNT), an assisted reproductive technology for producing cloned pigs. Nevertheless, the production efficiency of cloned animals, particularly pigs, is low^2,3^ and is affected by the type of donor cells, quality of oocytes, and type of recipient^4,5^. Moreover, the nuclear remodeling and reprogramming regimes used during the in vitro maturation (IVM) of oocytes affect the efficiency of the in vitro production (IVP) of embryos^6–8^. Overall, the quality of IVM-derived oocytes is the most important factor in determining the success rate of SCNT and the production of cloned porcine embryos, as the subsequent production is dependent on the developmental competence of pre-implantation embryos^9–11^. Additionally, the remodeling and reprogramming of donor nuclei are affected by the cytoplasmic factors of the recipient, where the level and content of cytoplasmic maturation are determining factors in the efficient production of cloned porcine embryos^12,13^.

As the oocyte progresses from the preantral to antral stages, it gradually develops the ability to resume meiosis. The resumption of meiosis begins with the dissolution of the nuclear envelope of the oocyte, known as “germinal vesicle breakdown” (GVBD) in the prophase of MI^14^. After GVBD and the completion of MI, the oocyte moves to meiosis II without an obvious S-phase and halts at metaphase II (MII) until fertilization. Several studies have investigated the mechanism behind meiotic arrest and have found that cyclic adenosine monophosphate (cAMP) as a cellular second messenger, plays a vital role in maintaining oocyte meiotic arrest^15,16^. Particularly in IVM-derived-porcine oocytes, nuclear maturation resumes following oocyte retrieval from the ovarian follicle due to an abrupt reduction in the level of cAMP and cGMP^17–20^. However, even after enhancing the developmental competence of oocytes through IVM, the quality of in vitro mature oocytes remains low compared with their in vivo counterparts^21,22^. Despite having undergone nuclear maturation, oocytes with incomplete cytoplasmic maturation were reported to inefficiently support the normal development of SCNT-derived embryos^23,24^. However, during IVM, the abrupt start of meiosis following retrieval of oocytes from the follicles leads to early or premature nuclear maturation that is manifested by the early extrusion of the first polar body, compared with that of in vivo oocytes^25–28^. Unstable meiotic progression results in the reduction of the time required for sufficient cytoplasmic maturation^29^. Furthermore, the gap junction communication (GJC) is responsible for maintaining meiotic arrest by attaining the required level of cAMP, which depends on the stage of nuclear maturation^30–33^. Therefore, the developmental competence of IVM-derived oocytes can be better achieved through synchronized cytoplasmic and nuclear maturation^34,35^.

This two-phase approach of IVM has shown improved developmental competence of the oocytes in different animal species, including bovine^36^, mice^37,38^, and sheep^39^. This improvement is due to the synchronized nuclear and cytoplasmic maturation achieved by delaying nuclear maturation, which ultimately leads to improved embryonic developmental following in vitro fertilization (IVF) and SCNT. Moreover, cAMP, acting as a second messenger, is known to play a critical role in the maintenance of meiotic arrest in mammalian oocytes^40–42^. Additionally, cAMP is synthesized in oocytes and follicular cells, including granulosa and cumulus cells. This protein enters the oocytes through the GJC and plays an active role in maintaining the meiotic arrest of the oocytes^43–46^.

To enhance the developmental competence of IVM oocytes, synchronized cytoplasmic and nuclear maturation is needed. This can be achieved by temporarily arresting the meiotic resumption of the nucleus^47,48^. The GJC is required to maintain a high level of cAMP, which inhibits the maturation-promoting factor and mitogen-activated protein kinase, thereby arresting meiosis^49,50^. In oocytes, cAMP is regulated by phosphodiesterase (PDE) and adenyl cyclase, which regulates the degradation and synthesis of cAMP^51–53^. In pigs and other animals, the developmental competence of IVM oocytes has been enhanced by regulating cAMP^54–57^. Similarly, cAMP has been reported to regulate meiotic arrest in bovine^58^ and mouse oocytes^59^.

Studies in pigs have demonstrated that using a single PDE inhibitor during IVM causes transient meiotic arrest in the oocytes^60–63^. For instance, a PDE3A inhibitor milrinone improved the quality of porcine embryos by synchronizing the nuclear and cytoplasmic maturation of oocytes with a low level of cytoplasmic development^63^. Furthermore, cilostamide-treated porcine oocytes showed improved developmental competence reflected by the higher quality of blastocysts from parthenogenetic activation (PA) and SCNT^64^. Similarly, cilostamide inhibited the activity of PDE3A in rodents and macaque oocytes^65,66^. Forskolin, another PDE3A inhibitor, successfully regulated cAMP in mouse^67,68^, rat^69,70^, dog^71^, and pig^72^ oocytes.

Despite progressive efforts, the in vitro competence of oocytes remains lower than that of their in vivo counterparts. On the other hand, rolipram as PDE4 inhibitor has showed a delaying effect on the spontaneous meiotic maturation during in vitro maturation through extended gap junctional communication between CCs and the oocyte, and subsequently improved cytoplasmic maturation and developmental potential of oocyte^73^. Therefore, IVM oocytes cannot adequately support the development of IVF and SCNT embryos.

We hypothesized that IVM conditions lack certain characteristics to mimic the in vivo conditions, particularly the synchronicity of nuclear and cytoplasmic maturation. Therefore, we aimed to investigate the effects of combined IVM treatment using PDE-3 and PDE-4 inhibitors, known to achieve the optimal cAMP level.

## Results

### Optimal concentration of rolipram and effective time for two-phase IVM in porcine oocytes

**Figure 1:**
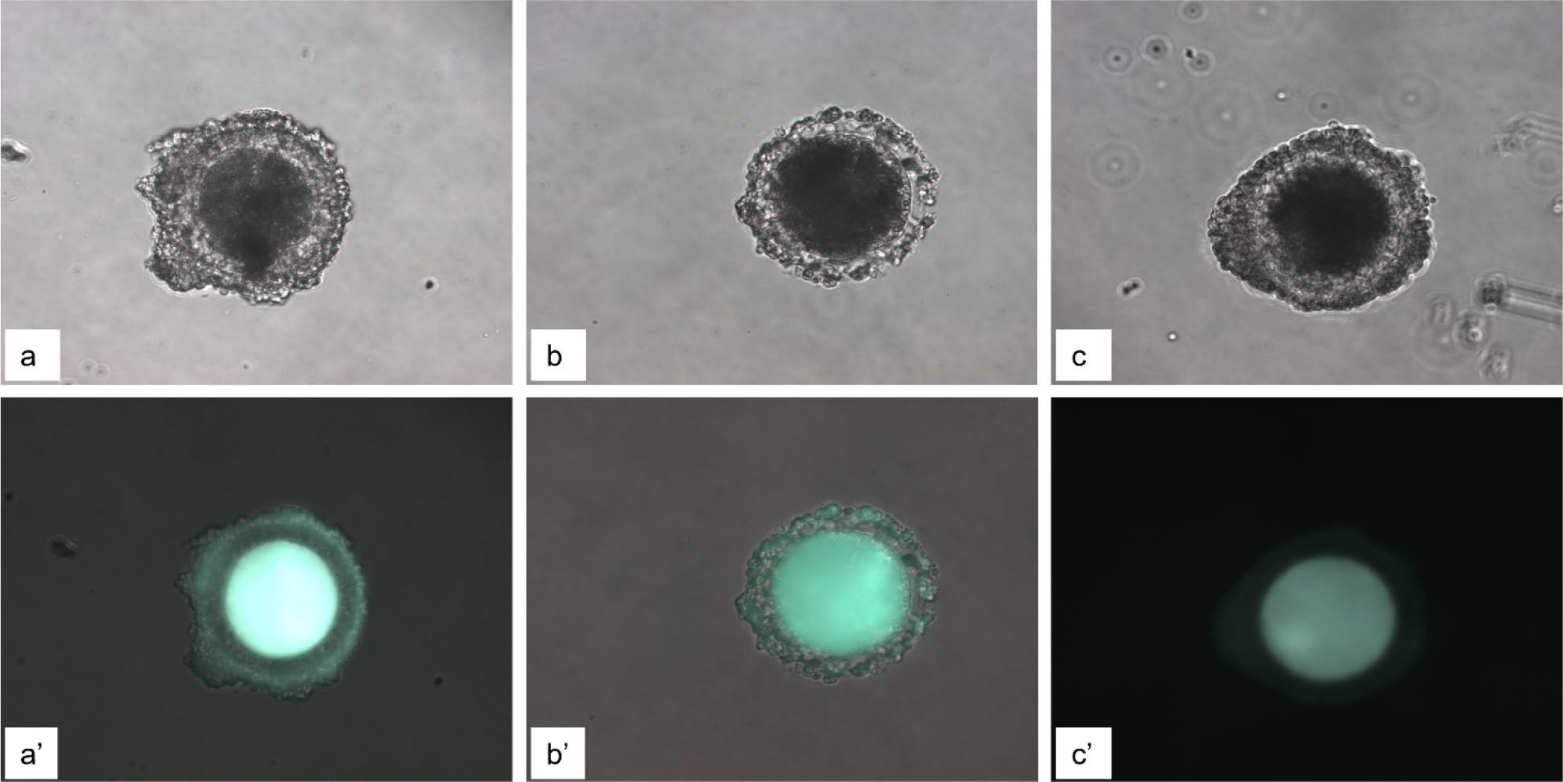
Images of the open status of functional GJCin porcine oocytes after pre-IVM rolipram–cilostamide treatment. Open functional GJC, almost all layers of cumulus cells are stained with lucifer yellow dye after intraoocyte microinjection (**a,a′**); partially open functional GJC, only one or two layers of cumulus cells are stained (**b,b′**); and closed functional GJC (**c,c′**), in bright light and UV, respectively.

To evaluate the effects of rolipram and cilostamide in maintaining functional GJC between the oocytes and their surrounding cells during pre-IVM, a Lucifer yellow dye transfer assay was conducted (Fig. 1). We used the reference time of 6 h for pre-IVM incubation based on previous findings^63^. Before IVM, only 5% of the COCs had closed GJC, whereas 80% had open GJC. Moreover, the percentage of oocytes with open GJC was 8.9 ± 0.8% and 10.5 ± 1.7%, 21.6 ± 3.5%, and 16.8 ± 4.1% in the control group and 25, 50, and 100 μM rolipram groups, respectively. The 50 μM rolipram group demonstrated a significant difference in the percentage of oocytes with open GJC from the other groups (P < 0.05). Similarly, the percentage of oocytes with partially open GJC was 29.3 ± 5.0%, 28.3 ± 2.5%, 40.7 ± 3.9%, and 31.2 ± 7.0% in the corresponding groups. Finally, the respective percentages of oocytes with closed GJC were 61.8 ± 8.5%, 59.1 ± 8.3%, 37.7 ± 13.3%, and 52.0 ± 13.8%, with a significant difference (P < 0.05) between the treated and control groups (Table 1). Therefore, we determined the optimal concentration of rolipram for maintaining functional GJC in porcine oocytes to be 50 μM. We then determined the effective time for two-phase IVM in porcine oocytes using rolipram and cilostamide, inhibiting cAMP in cumulus cells and intraoocytes, respectively.

**Table 1:** The effect of different concentrations of rolipram in maintaining functional gap junctional communications for first 6 h in porcine oocytes during in vitro maturation.

Concentration (μM)	No. of oocytes by status of functional gap junctional communications		
	Open (%)	Partially opened (%)	Closed (%)
Control (0)	16 (8.9 ± 0.8)^a^	52 (29.3 ± 5.0)	110 (61.8 ± 8.5)^a^
25	12 (10.5 ± 1.7)^a,b^	56 (28.3 ± 2.5)	117 (59.1 ± 8.3)^a,b^
50	43 (21.6 ± 3.5)^b^	81 (40.7 ± 3.9)	75 (37.7 ± 13.3)^b^
100	29 (16.8 ± 4.1)^a^	54 (31.2 ± 7.0)	90 (52.0 ± 13.8)^a,b^

Values are listed as the mean ± SEM.

Means in the same column with different superscript letters were significantly different (P < 0.05).

**Figure 2:**
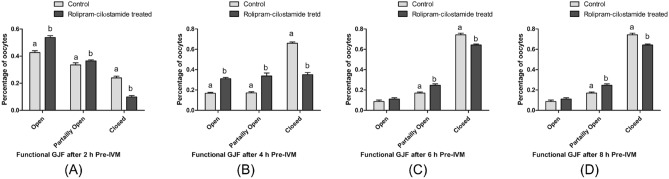
The optimal time for pre-IVM incubation of porcine oocytes with rolipram–cilostamide treatments. Percentage of oocytes with open, partially open, and closed GJC after 2 (**A**), 4 (**B**), 6 (**C**), and 8 (**D**) h of pre-IVM incubation. A significant difference (P < 0.05) was observed between the control and rolipram–cilostamide-treated groups for open, partially open, and closed GJC at 2 and 4 h but not 6 or 8 h. The percentage of oocytes with open and partially open GJC was less than 10%. As seen in (**A,B**), after 4 h of pre-IVM incubation, rolipram–cilostamide treatment significantly affected functional GJC status, and this period was determined to be the optimal time for the two-phase IVM of porcine oocytes using rolipram and cilostamide.

The optimal concentrations of rolipram and cilostamide were determined based on the maintenance of functional GJC during pre-IVM incubation. The percentage of oocytes with open, partially open, and closed GJC for the first 2 h of pre-IVM incubation was 42.6 ± 0.21%, 33.5 ± 0.17%, and 23.9 ± 0.12%, respectively, in the control group versus 53.7 ± 0.27%, 36.4 ± 0.18%, and 9.9 ± 0.05%, respectively, in the rolipram–cilostamide-treated group (P < 0.05). Similarly, the percentage of oocytes with open, partially open, and closed GJC for the first 4 h of pre-IVM incubation was 16.9 ± 0.08, 17.2 ± 0.09, and 66.0 ± 0.33%, respectively, in the control group versus 31.2 ± 0.16, 33.8 ± 0.17, and 35.0 ± 0.18%, respectively, in the rolipram–cilostamide-treated group (P < 0.05) (Fig. 2).

**Figure 3:**
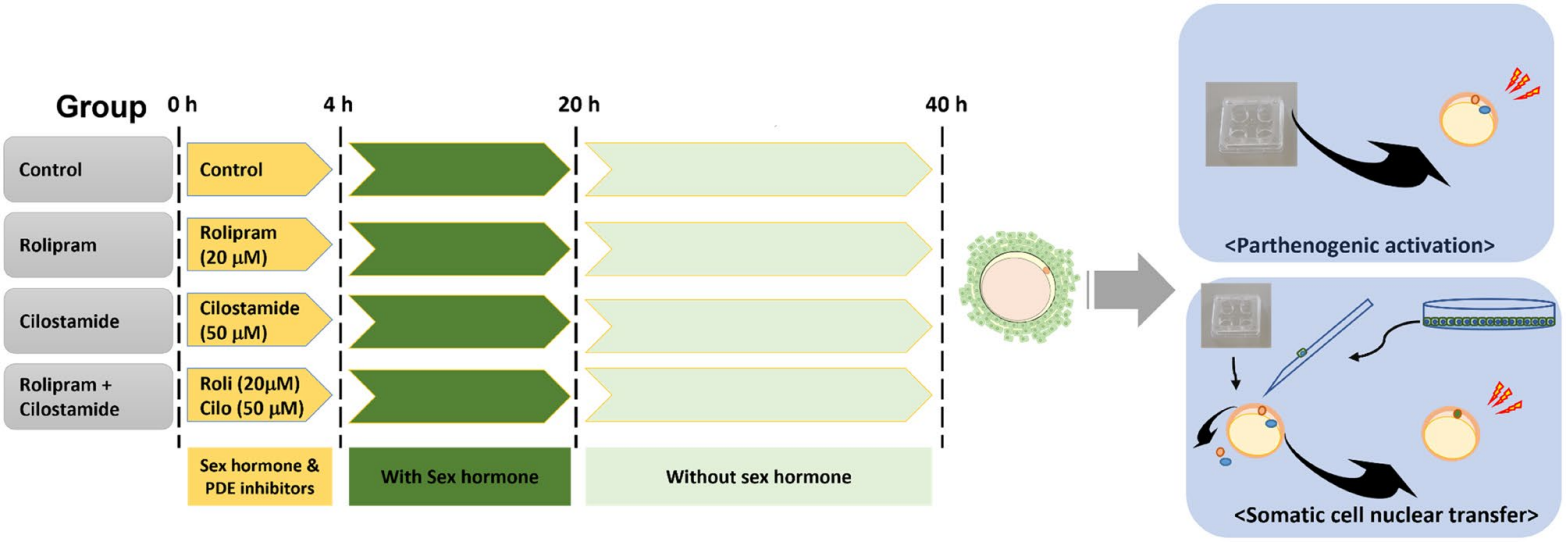
Schematic diagram of the experimental design for two-phase IVM systems using the PDE inhibitors rolipram and cilostamide, with embryonic development after parthenogenesis and SCNT.

Conversely, after 6 h of pre-IVM incubation, the percentage of oocytes with open and partially open GJC were found to be lower as compared with that after4 h of pre-IVM incubation (from 31.2% and 33.8% to 11.1% and 24.6%, respectively) (Fig. 2). Furthermore, after 6 h of pre-IVM, the GJC status of most oocytes changed to closed (64.3% versus 35.0% after 4 h of pre-IVM incubation) (Fig. 2). Accordingly, we determined the effective time for maintaining functional GJC using rolipram and cilostamide in porcine oocytes to be 4 h of pre-IVM incubation. Accounting for sex hormones and the need for the synchronization of cytoplasmic and nuclear maturation, we designed a three-stage IVM for porcine oocytes. First, oocytes were treated with sex hormones and PDE inhibitors (4 h of incubation), followed by incubation for 16 h with sex hormones, and then 20 h of incubation without hormones (Fig. 3).

### Effects of two-phase IVM on GSH and ROS level of IVM oocytes

**Figure 4:**
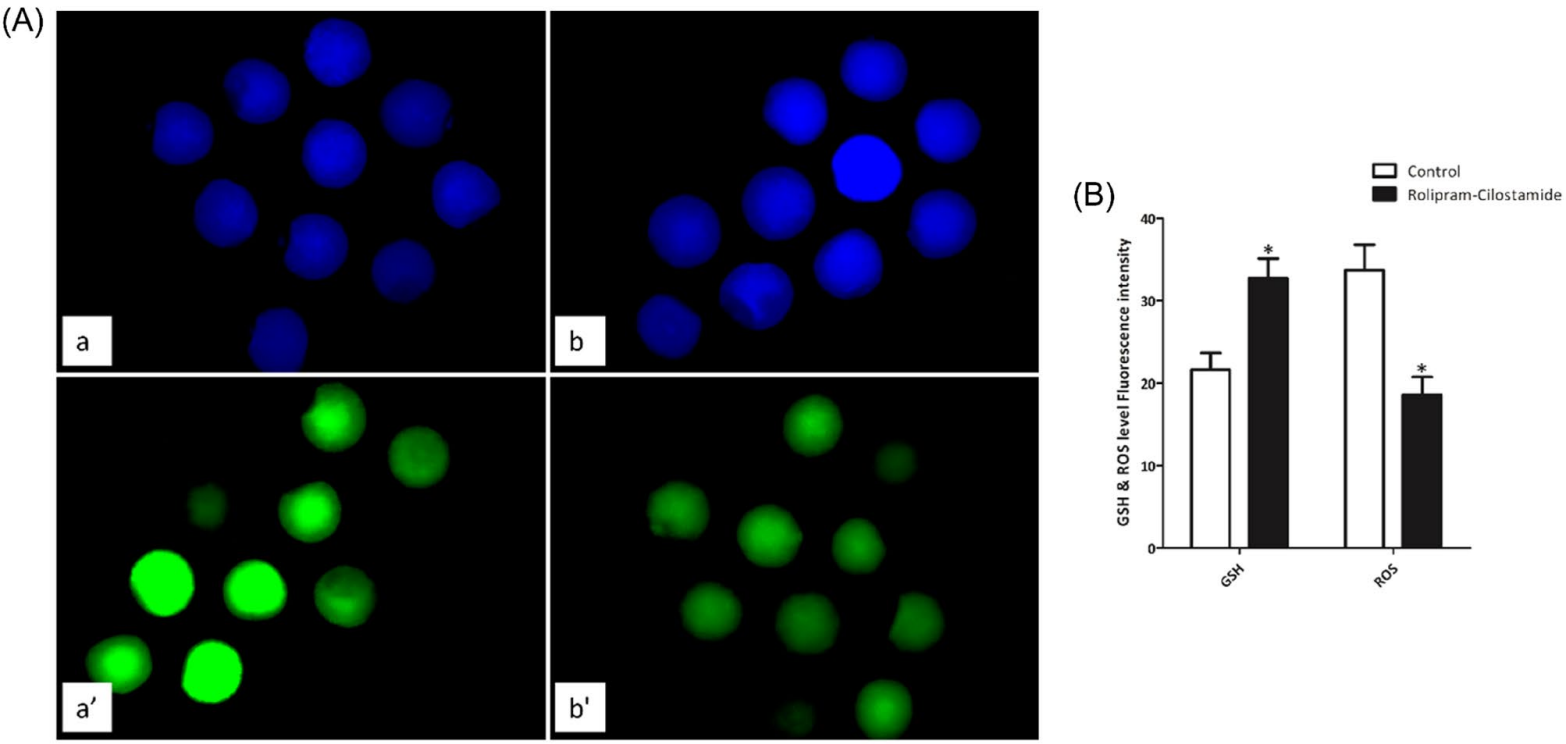
Epifluorescence photomicrographic images of IVM porcine oocytes. (**A**) Oocytes were stained with (a,b) Cell Tracker Blue and (a′,b′) 2′,7′-dichlorodihydrofluorescein diacetate (H2DCFDA) to detect intracellular GSH and ROS levels of control (a,a′) and rolipram–cilostamide-treated oocytes (b,b′). (**B**) Effects of treatments on intracellular levels of IVM porcine oocytes (GSH and ROS levels in each group), bars with *indicate significant differences (P < 0.05). To measure the GSH and ROS levels, at least 40 oocytes were used for each experiment, which was replicated 8 times.

Following IVM, oocytes were denuded, and those with the first polar bodies were used to measure GSH and ROS levels. The GSH levels were significantly higher in the rolipram–cilostamide-treated groups than in the control group, while the ROS levels were found to be significantly lower in the rolipram–cilostamide-treated oocytes than in the control group oocytes (P < 0.05) (Fig. 4).

### Effect of two-phase IVM on the level of cytoplasmic maturation

**Figure 5:**
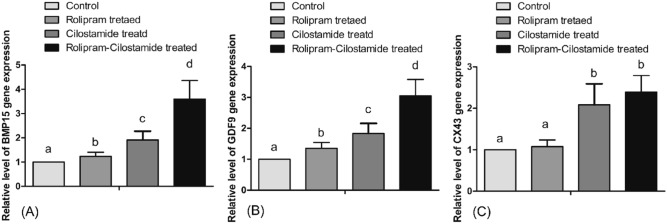
The relative level of gene expression on quantitative analysis of mRNA transcripts by real-time PCR in control, rolipram, cilostamide, and rolipram–cilostamide-treated porcine oocytes during IVM. At least 80 oocytes were obtained per sample and 8 biological replications were performed. Relative expression of BMP15 (**A**) and GDF9 (**B**) to determine the level of cytoplasmic maturation were statistically significantly different among groups. (**C**) Relative level of CX43 gene expression for level of functional GJC is significantly different between cilostamide and rolipram–cilostamide-treated groups versus the control and rolipram-treated groups (P < 0.05). Values in columns with different superscript letters are significantly different. Control samples were set as arbitrary units, and the target genes were expressed as a fold-change of the corresponding control relative to the house keeping gene (β-actin).

The purpose of two-phase IVM is to temporarily inhibit the abrupt resumption of nuclear maturation following retrieval, as cytoplasmic maturation is inadequate to support subsequent embryonic development. The level of cytoplasmic maturation in IVM oocytes was determined by assessing the relative expression level of genes associated with cytoplasmic maturation. The expression level of *GDF9* and *BMP15* were significantly different between the rolipram–cilostamide-treated group and other groups (Fig. 5).

### Effect of two-phase IVM on cleavage and blastocyst development in PA embryos

To evaluate the effects of two-phase IVM on the developmental competence of PA embryos, we cultured the IVM oocytes after activation. The cleavage rate was not significantly different among the control, rolipram-, cilostamide-, and rolipram–cilostamide-treated groups, whereas blastocyst formation rates were significantly higher in the rolipram–cilostamide group than in other groups (Table 2). The cleavage rate in the control, rolipram-, cilostamide-, and rolipram–cilostamide-treated groups was 71.5%, 72.6%, 72.9%, and 76.0%, respectively. Therefore, the rolipram–cilostamide-treated group showed a significantly higher cleavage rate than the control group, while no significant difference was observed between the rolipram–cilostamide-treated group and the other treatment groups. Moreover, the blastocyst development rate was significantly different between the rolipram–cilostamide-treated group and the other groups (27.2%, 28.1%, 30.0%, and 35.5% in the control, rolipram-, cilostamide-, and rolipram–cilostamide-treated groups, respectively). Finally, the blastocyst development rate did not differ significantly between the rolipram group and the control or cilostamide groups.

**Table 2:** Effect of two-phase treatment of rolipram and cilostamide in first 4 h of in vitro maturation on development of parthenogenically activated porcine embryos.

Treatments	No. of embryos		
	Cultured	Cleaved (%)	Develop to blastocyst (%)
Control	316	226 (71.5 ± 1.5)^a^	86 (27.2 ± 0.7)^a^
Rolipram (50 µM)	317	230 (72.6 ± 1.4)^a,b^	89 (28.1 ± 0.5)^a,b^
Cilostamide (20 µM)	317	231 (72.9 ± 1.8)^a,b^	95 (30.0 ± 0.7)^b,c^
Rolipram (50 µM) + cilostamide (20 µM)	313	238 (76.0 ± 0.7)^b,c^	111 (35.5 ± 0.6)^d^

Values are listed as the mean ± SEM.

Means in the same column with different superscript letters were significantly different (P < 0.05).

### Effect of two-phase IVM on cleavage and blastocyst development rate in SCNT embryos

To evaluate the effects of two-phase IVM on the developmental competence of SCNT-derived embryos, the cleavage and blastocyst development rates were determined after culturing reconstructed oocytes for 2 and 7 days, respectively, in a PZM-5 medium. The cleavage rate was significantly different between the control and rolipram–cilostamide-treated groups (69.1% versus 77.0%, respectively). Similarly, the blastocyst development rate was significantly higher in the rolipram–cilostamide-treated group than in the control group (27.3%versus 19.5%, respectively; P = 0.000; Table 3).

**Table 3:** Effect of two-phase in vitro maturation using rolipram and cilostamide for the first 4 h on development of porcine transgenic cloned embryos.

Treatments	No. of embryos		
	Cultured	Cleaved (% ± SEM)	Develop to blastocyst (% ± SEM)
Control	333	230 (69.1 ± 1.1)^a^	65 (19.5 ± 0.6)^a^
Rolipram (50 µM) + cilostamide (20 µM)	330	254 (77.0 ± 1.6)^b^	90 (27.3 ± 1.1)^b^

Values are listed as the mean ± SEM.

Means in the same column with different superscript letters were significantly different (P < 0.05).

### Pre-implantation developmental competence of two-phase blastocysts

**Figure 6:**
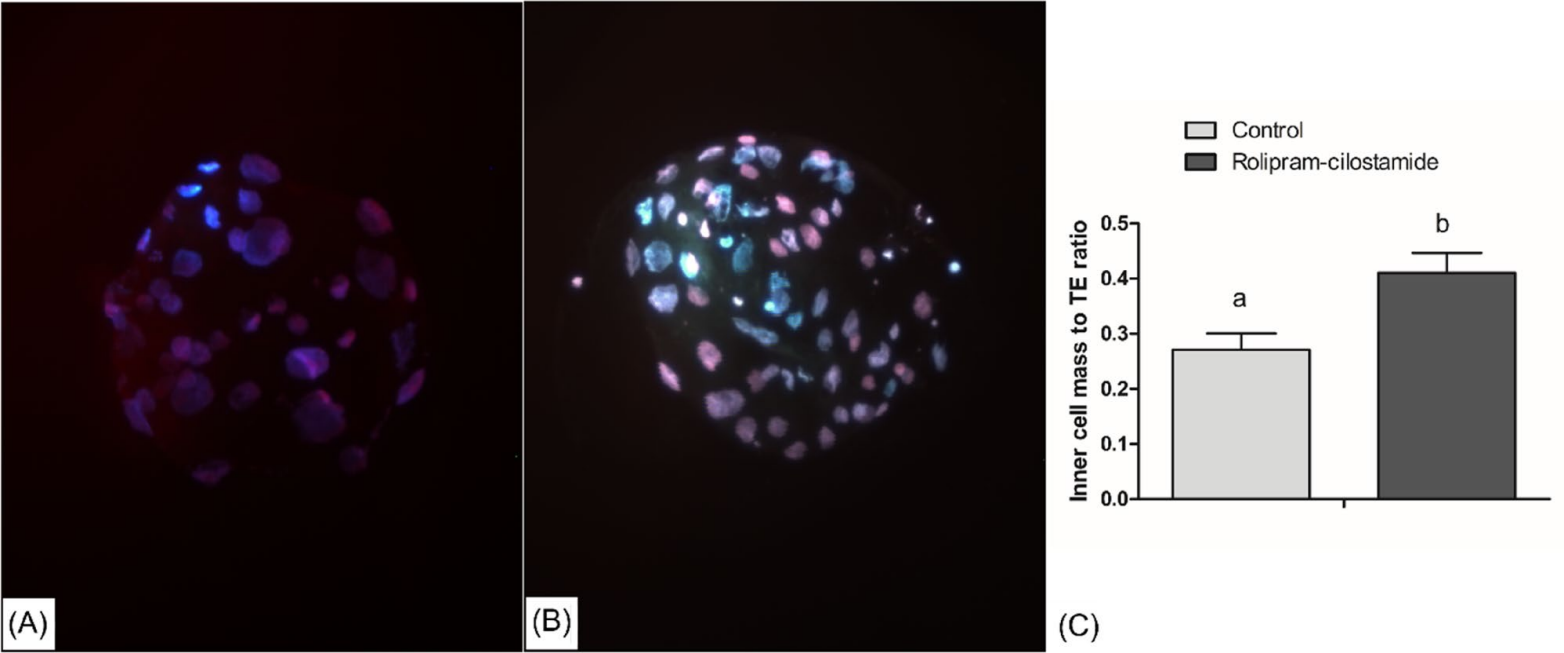
Effect of combined two-phase IVM with rolipram and cilostamide on the embryonic development of transgenic cloned porcine embryos. Representative images of blastocysts developed from oocytes matured in control (**A**) and rolipram–cilostamide (**B**) during IVM. At least 68 blastocysts were stained from each group. (**C**) Inner cell mass to TE ratio in differential staining. The inner cell mass to TE ratio in blastocysts from two-phase IVM differed significantly from that from the control (0.399 vs 0.269, respectively, P < 0.05). The nuclei of ICM and TE cells were stained with Hoechst (blue) and PI (red) dye, respectively. The data are from at least three independent experiments, and the values represent the mean ± SEM.

We evaluated the pre-implantation developmental competence of embryos derived from the two-phase IVM oocytes using a differential staining technique to determine the total cell count, ICM, and TE ratio. The results showed a significant difference between the rolipram–cilostamide-treated and control groups in terms of total cell count, ICM, and TE ratio, respectively. Specifically, the ICM (10.7 ± 1.31 versus 6.8 ± 0.78) and TE ratio (25.2 ± 0.83 versus 26.8 ± 1.63) were significantly higher in the rolipram–cilostamide-treated group compared to the control group (Fig. 6). Moreover, the ICM to TE ratio was significantly higher in the embryos derived from the rolipram–cilostamide-treated oocytes than in the control group oocytes (0.269 versus 0.399, respectively).

### Relative level of gene expression

**Figure 7:**
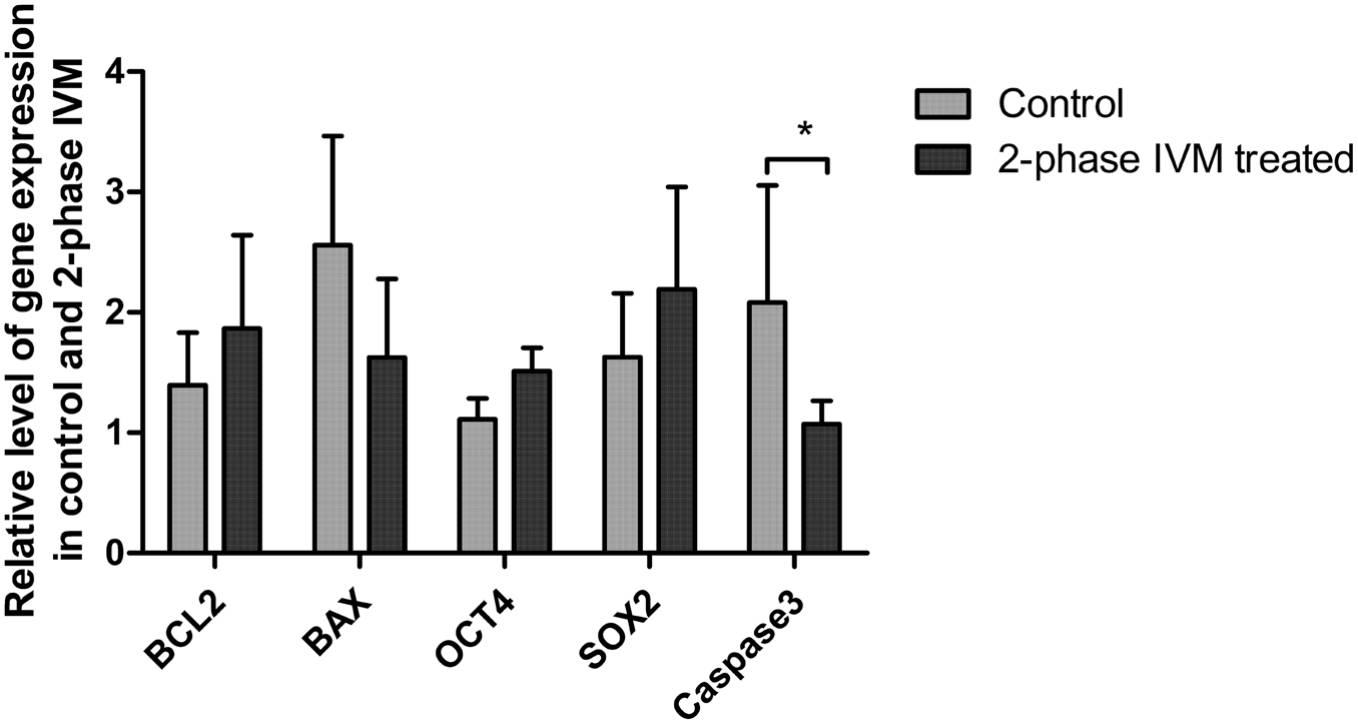
Relative quantitative analysis of mRNA transcript expression by real-time PCR in control and two-phase IVM porcine oocyte blastocysts. At least 5 blastocysts were obtained per sample and 8 biological replications were performed. Control samples were set as arbitrary units, and the target genes were expressed as the fold-change of the corresponding control relative to the house keeping gene (β-actin). *Indicates a significant difference at P < 0.05.

The effect of two-phase IVM was evaluated according to the relative level of mRNA expression for nuclear modeling and reprogramming in SCNT blastocysts (Fig. 7). Notably, the expression of the pro-apoptotic gene (caspase 3) was found to be statistically significantly different between control and two-phase treated groups (P < 0.05). Moreover, there is a tendency to reduce the expression of pro-apoptotic gene *BAX* while the expressions of BCL2, Oct4, and SOX2 tended to be increased in the two-phase treated groups (P < 0.1)*.*

## Discussion

During the IVM of mammalian oocytes, cAMP modulators demonstrate significant improvement in terms of oocyte developmental competence to support subsequent embryonic development. Specifically, cAMP modulators, including milrinone, cilostamide, and 3-isobutyl-1-methylxanthine, have positive effects on the developmental competence of oocytes. Improving oocyte competence enhances later embryonic development to the blastocyst stage^74^. To do so, the optimal level of cAMP must be achieved to regulate adenyl cyclase activators and/or PDE inhibitors that cause temporary meiotic arrest without permanently impairing meiotic progression. Previous reports have demonstrated that cytoplasmic maturation can be synchronized with nuclear maturation using PDE inhibitors either during IVM or pre-IVM^75–77^.

Several studies have reported that cAMP modulators enhance the developmental competence of porcine oocytes after IVF and SCNT. The use of cAMP modulators during IVM results in the proper cytoplasmic maturation of oocytes to support the subsequent embryonic development^78–80^. Another study reported that cAMP modulators, including cilostamide and forskolin, effectively cause meiotic arrest in the oocytes following pre-IVM for 24 h^32^. However, cAMP modulators may negatively regulate the meiosis of oocytes, particularly when higher concentrations are used or oocytes are exposed for a longer duration^81^. Similar findings were also reported during the IVM of bovine oocytes^58^. However, our previous study showed that the use of higher concentrations of milrinone during IVM of porcine oocytes had no negative effects^63^. In the current study, we observed that higher concentrations of PDE inhibitors have non-beneficial effects, which could be due to permanent or prolonged meiotic arrest, which negatively affects IVM.

The current study demonstrated that pre-IVM treatment of rolipram (using its optimal concentration) significantly affected the developmental competence of IVM oocytes. Rolipram affects the functional GJC required for attaining higher levels of cAMP to induce temporary meiotic arrest in porcine oocytes. Similarly, rolipram treatment induced meiotic arrest in rat oocytes^82^ but failed to induce meiotic arrest in bovine oocytes^83^.

Various studies have reported a higher level of cytoplasmic maturation during the two-phase approach compared to the single-phase PDE inhibitor approach. The non-specific PDE inhibitor IBMX inhibited both cumulus cell and oocyte PDEs, whereas the PDE3-specific inhibitor cilostamide inhibited only intraoocyte oocyte PDEs^84^. In contrast, PDE4 inhibitors was reported to affect only cAMP levels in cumulus cells^48,80^. In the current study, we proposed using a combination of PDE3 and PDE4 inhibitors to attain optimal synchronization of cytoplasmic and nuclear maturation. This two-phase IVM procedure significantly affected the GJC and subsequent developmental competence of porcine oocytes. Specifically, the two-phase procedure effectively maintained the GJC and the level of cytoplasmic maturation in the porcine oocytes compared to the single-phase PDE-treated groups and the control group. These findings are consistent with previous reports of improved competence of bovine oocytes in the presence of invasive adenyl cyclase^85,86^.

Two-phase IVM approaches intend to improve the developmental competence of IVM oocytes through the spontaneous regulation of nuclear maturation and oxidative stress^84^. In the current study, our two-phase IVM approach enabled synchronized cytoplasmic and nuclear maturation, which is evident by the significantly higher relative expression of the genes associated with cytoplasmic maturation, including *BMP15* and *GDF9*. Similar findings were reported in other studies where the cytoplasmic maturation of porcine oocytes was confirmed through *BMP15* and *GDF9* upregulation^87,88^. The findings of the current study are also consistent with the study by Funahashi and his team, who reported that the two-phase maturation of the germinal vesicle of porcine oocytes during IVM is responsible for the subsequent developmental competence^89^. Furthermore, the higher level of GSH and lower level of ROS after two-phase IVM were consistent with a previous study by Park et al.^32^ and contributed to improving the developmental competence of the oocytes.

Our results demonstrated that two-phase IVM yielded better oocyte competence. Moreover, the two-phase IVM approach resulted in a significantly higher blastocyst formation of PA embryos than that of other groups. Several studies have reported similar findings in sheep^39^, rats^90^, bovine^91^, and pigs^71^. This improved developmental competence of PA embryos was likely due to the synchronized maturation of the nucleus and cytoplasm to support the subsequent embryonic development. Similar findings were reported in mice^92^ and other mammals^93^. Moreover, the relative reductions in the expression of *BAX*^94^ and Caspase-3^95^ in two-phase as compared to the control group were a good indication of the development competence of embryos from two-phase treated group (Fig. 7). The relative level of the expression of anti-apoptotic genes^94^ and nuclear reprograming related gene *OCT4* and *SOX2*^94,96^ were well recognized genes as indicator of developmental competence of in vitro produced porcine embryos.

In the cloned embryos derived from oocytes subjected to two-phase IVM, both the cleavage rate and blastocyst formation rate were significantly higher than those derived from the control oocytes. Another study showed similar findings using cilostamide, which significantly improved the formation of porcine SCNT blastocysts^71^. In bovine embryos, the two-phase IVM approach led to significant variation in the level of cytoplasmic maturation and subsequent blastocyst formation^73^. The results of the current study revealed that meiotic progression could be reversibly attenuated through the combination of PDE3 and PDE4 inhibitors to synchronize nuclear and cytoplasmic maturation in porcine oocytes.

Finally, the evaluation of the blastocysts for pre-implantation development by differential staining and tunnel assay also showed that two-phase IVM significantly improved the developmental competence in porcine blastocysts, consistent with the 2016 findings by Park et al.^32,63^. Our findings also support the hypothesis that a higher total cell count and ICM are indicators of the developmental competence of blastocysts in various species of mammals^97,98^. In conclusion, we found that two-phase IVM of porcine oocytes can be attained through the combination of the PDE3 and PDE4 inhibitors (50 µM rolipram and 20 µM cilostamide) for 4 h pre-IVM. This two-phase IVM played a significant role in synchronizing the cytoplasmic and nuclear maturation, which is pivotal in nuclear remodeling and reprogramming, particularly in SCNT. We suggest further experiments be conducted for the wide-scale application of two-phase IVM in porcine oocytes.

## Conclusions

Rolipram and cilostamide application during pre IVM as phosphodiesterase inhibitor 4 and 3, respectively, had improved the functioned GJC in porcine oocyte as indicated by enhancing the in vitro matured oocyte competence in terms of cytoplasm maturation and supported the subsequent embryonic development. We also showed that the combined treatment of rolipram and cilostamide attained the relatively better preimplantation development competence of multigene modified cloned porcine embryos.

## Materials and methods

### Culture media

Sigma-Aldrich (St. Louis, MO, USA) is source of all chemicals and reagents used unless stated. Cilostamide and rolipram stock solutions were prepared in dimethyl sulfoxide and aliquots were prepared during pre-IVM. Cilostamide was used at a concentration of 20 µM^99^ based on previous studies, and we determined the optimal concentration and time of rolipram for pre-IVM as there were no previous study. The medium used for IVM tissue culture medium-199 (Invitrogen, Carlsbad, CA, USA), which were supplemented with 10% porcine follicular fluid (v/v), 0.6 mM cysteine, 0.91 mM pyruvate, 75 mg/mL kanamycin, and 1 mg/mL insulin. The in vitro culture (IVC) medium used for embryonic in vitro culture was porcine zygote medium-5 (PZM5; IFP, Yamagata, Japan).

### Oocyte collection and in vitro maturation

Oocyte IVM was performed following our laboratory protocols as previously described^100^. Ovaries from gilts. Oocytes with sizes of 3–8 mm in diameter were aspirated and aspirates were washed as mentioned in our previous study^101^.

### Measurement of GJC

The functional GJC was evaluated through the intraoocyte microinjection of Lucifer yellow, whose dissemination level to cumulus cells was evaluated^102^. The classification of the functional state of JGC were open, partially open, or closed^103^.

### Intraoocyte GSH and ROS levels measurement

Glutathione (GSH) and reactive oxygen species (ROS) levels were measured as described previously^104–106^. 2′,7′-dichlorodihydrofluorescein diacetate (H2DCFDA; Invitrogen), on the bases of the intensity of fluorescence to measure the ROS level. The GSH level determination was performed using Cell Tracker™ Blue (CMF2HC). About ten to thirteen oocytes from each treatment group (control, rolipram treated, cilostamide treated and combination of rolipram and cilostamide treated) were incubated in TLH-PVA supplemented with 10 mM H2DCFDA and 10 mM CMF2HC for 30 min in the dark. Then, the oocytes were washed Dulbecco’s Phosphate Buffered Saline (dPBS) (Invitrogen) containing 0.1% (w/v) PVA. The GSH and ROS levels were evaluated in different groups using epifluorescence microscope (Leica DM IRB; Leica Microsystems) and fluorescence intensity evaluation is performed using ImageJ software (version 1.41; National Institutes of Health, Bethesda, MD, USA)^101^.

### Preparation of donor cells

Cells with genes modified for human immune reactivity, i.e., alpha-1,3-galactosyltransferase (*GGTA1*), cytidine monophosphate-N-acetylneuraminic acid hydroxylase (*CMAH*), and alpha 1,3-galactosyltransferase 2 (*A3GALT2*) triple knockout (GGTA1 + CMAH + iGb3S TKO) porcine cells were used as donor cells^107^. The cells were seeded in a four-well plate and cultured for at least 2 days in dPBS (Invitrogen) with 15% (v/v) fetal bovine serum and 75 mg/mL kanamycin. Cells followed until a complete monolayer formed and passage 3 or 4 were used as donor cells. A single-cell suspension was prepared by trypsinizing cultured cells and resuspending in TLH containing 0.4% (w/v) bovine serum albumin (BSA) (TLH–BSA) before nuclear transfer.

### SCNT, PA, and embryo culture

The COCs were cultured and incubated overnight in a pre-treated IVM medium. After 40–42 h of IVM, matured oocytes were denuded in 0.1% (w/v) hyaluronidase in a hormone-free IVM medium. Invitro procedures were performed in calcium-free TLH–BSA with 5 mg/mL cytochalasin B. Before manipulation, oocytes were incubated for at least 30 min in a manipulation medium containing 5 µg/mL Hoechst 33342. Following washing, the oocytes were placed into a droplet of manipulation medium covered with mineral oil. Enucleation was performed by aspirating the first polar body by using a 17-mm beveled glass pipette. Enucleation was confirmed under an epifluorescence microscope (TE300; Nikon, Tokyo, Japan). Then, the oocytes were transferred to another medium for cell injection, and around 20 cells were aspirated into pipette; and one cell was inserted as donner cell^108^. Then, fusion was performed using a BTX 2001 Electro-cell Manipulator. The activated embryos of both PA and SCNT were treated either 7.5 µg/mL cytochalasin B and 0.4 mg/mL demecolcine, respectively then transferred into 25-µL IVC. Cleavage and blastocyst development were evaluated following two and seven days of culturing, respectively, with the day of SCNT or PA designated as Day 0.

### Quantitative real-time PCR (q-PCR)

We extracted total RNA from Day 7 blastocysts RNeasy Micro Kit (QIAGEN, Hilden, Germany), according to the manufacturer’s protocol. The primer sequences listed in Table 4 were used to evaluate the abundance of the transcripts.

**Table 4:** List of primers used for the relative level of gene expression for pre-implantation developmental competence in SCNT blastocysts, cytoplasm maturation, and functional GJC in IVM oocytes.

Genes	Primers (5ʹ–3ʹ)	Product size (bp)	Annealing temp (°C)	Accession number
*β-actin*	F: CCC TGG AGAAGAGCTACGAG R: TCCTTCCTGATGTCCACGTC	172	55	NC_010445.4
*CX43*	F: GGAGCAGCCATTGAAATAAGC R: GGAGCAGCCATTGAAATAAGC	232	60	NM_001244212.1
*SOX2*	F: TACAGCATGATGCAGGAC R: GAGCTGGTCATGGAGTTG	128	55	NM_001123197
*OCT4*	F: GCCAGAAGGGCAAACGAT R: AGGGTGGTGAAGTGAGGG	154	57	NM_001113060
*BCL2*	F: TTCTCTCGTCGCTACCGC R: CCAGTTCACCCCATCCCT	123	55	XM_021099593
*BAX*	F: CCT TTT GCT TCA GGG TTT CA R: ATC CTC TGC AGC TCC ATG TT	110	55	XM_003127290
*Caspase 3*	F: TTTCTAAAGAAGACCATAGCAAAA R: CTGAATTATGAAAAGTTTGGGTTTG	176	54	NC_010457.5
*GDF9*	F: GAGCCTTGGTCCTTGCTG R: AAGCTCTGGAGTCTGGCT	158	59	NC_010444.4
*BMP15*	F: CTGGTGAGGCCATTGGTTAA R: GCAATTAGATAGTCAGCTAA	170	58	NC_010461.5

### Differential staining in SCNT blastocysts

We performed differential staining to determine blastocyst competence using the total cell number. The inner cell mass (ICM) to trophectoderm (TE) ratio was used as indicator of pre-implantation embryos development competence, as described in our previous reports^104^. Briefly, diluted Triton X-100 in PBS (T solution) at concentration of 0.1% (v/v), and propidium iodide (PI) (25 μg/mL) and Hoechst 33342 solutions (H solution) (5 μg/mL) were used for the staining. The staining followed by placing blastocysts in H solution first (40 min) then in T solution (1 min) at room temperature and then brief period (30–40 s) in PI solution.

### Experimental design

For pre-IVM treatment, immature COCs were either untreated (control) or treated with 50 µM rolipram, 20 µM cilostamide, or both. Then, the level of cytoplasm maturation, ROS, and GSH of the oocytes and the status of GJC in the pre-IVM oocytes were assessed. Furthermore, the developmental competence of PA and SCNT embryos was assessed. The duration of pre-IVM in this study was determined according to the GJC and IVM oocyte competence, and the starting reference value was taken from pigs^109^ and humans^99^.

In experiment 1, the effects of rolipram–cilostamide treatment on GJC during pre-IVM, intraoocyte ROS and GSH levels, and embryonic development after PA were examined. Subsequently, in experiment 2, the effects of rolipram–cilostamide treatment on cytoplasm maturation status, as an indication of the synchrony of nucleus and cytoplasm maturation, were examined in IVM oocytes. In experiment 3, the effects of rolipram–cilostamide combination treatment on the developmental competence of oocytes were compared with those of rolipram and cilostamide monotreatment. In experiment 4, we assessed the pre-implantation developmental competence of PA and SCNT blastocysts (Fig. 3).

### Statistical analysis

At least eight replicates of each experiment were performed, and the values were compared as the mean ± standard error of the mean (SEM). SPSS version 22 (SPSS 22.0; IBM, Armonk, New York, USA) was used for the statistical analyses. Moreover, in case of two groups, independent sample t-test was used, while univariate analysis of variance (ANOVA) with Tukey’s multiple comparison tests were used to determine the significant differences among the four experimental groups. P-values < 0.05 were considered significantly different among the experimental groups.

## Data Availability

The data sets used during the current study are available from the corresponding author on reasonable request.

## Abbreviations

IVM: In vitro maturation
PA: Parthenogenic activation
SCNT: Somatic cell nuclear transfer
dPBS: Dulbecco’s phosphate buffered saline
cAMP: Cyclic adenine monophosphate
BMP15: Bone morphogenetic protein 15
cGMP: Cyclic guanine monophosphate
GDF9: Growth development factor 9
IVP: In vitro production
GJC: Gap junction communication
IVF: In vitro fertilization
PDE: Phosphodiesterase
IVC: In vitro culture
GSH: Glutathione
ROS: Reactive oxygen species
BSA: Bovine serum albumin
COC: Cumulus oocyte complexes
SEM: Standard error of the mean
PZM: Porcine zygote media
ICM: Inner cell mass
TE: Trophectoderm
mRNA: Messenger ribonucleic acid
